# Plasma Inflammatory Biomarkers Associated with Advanced Liver Fibrosis in HIV–HCV-Coinfected Individuals

**DOI:** 10.3390/ijerph17249474

**Published:** 2020-12-17

**Authors:** Xiaochen Chen, Xing Liu, Song Duan, Renhai Tang, Sujuan Zhou, Runhua Ye, Yuecheng Yang, Jibao Wang, Shitang Yao, Na He

**Affiliations:** 1 Department of Epidemiology, School of Public Health and the Key Laboratory of Public Health Safety of Ministry of Education, Fudan University, Shanghai 200032, China; xcchen16@fudan.edu.cn (X.C.); liuxing@fudan.edu.cn (X.L.); sjzhou@fudan.edu.cn (S.Z.); 2 Key Laboratory of Health Technology Assessment of Ministry of Health, Fudan University, Shanghai 200032, China; 3 Dehong Prefecture Center for Disease Control and Prevention, Mangshi 678400, China; dhduansong@sina.com.cn (S.D.); trh1020@163.com (R.T.); dhcdcyrh@163.com (R.Y.); yyc0605@126.com (Y.Y.); jbwang.aids@163.com (J.W.); yaoshitang@sina.com (S.Y.)

**Keywords:** HIV, HCV, liver fibrosis, microbial translocation, inflammation

## Abstract

Background: HIV and HCV coinfection leads to accelerated liver fibrosis, in which microbial translocation and systemic inflammation might play important roles. Objective: This study aimed to provide an extensive profile of the plasma microbial translocation and inflammation biomarkers associated with advanced liver fibrosis among HIV–HCV-coinfected patients. Methods: This cross-sectional study recruited 343 HIV–HCV-coinfected patients on combination antiretroviral therapy (cART) from a rural prefecture of Yunnan province in Southwest China. The plasma concentrations of sCD14 and 27 cytokines and chemokines were assayed and compared against advanced or mild levels of liver fibrosis. Results: Of the 343 HIV–HCV-coinfected patients, 188 (54.8%) had severe or advanced liver fibrosis (FIB-4 > 3.25). The patients with advanced liver fibrosis (FIB-4 > 3.25 vs. FIB-4 ≤ 3.25) had higher plasma levels of interleukin (IL)-1β, IL-6, IL-7, IL-9, IL-12, IL-15, IL-17, granulocyte macrophage colony stimulating factor (GM-CSF), Interferon-γ (IFN-γ), tumor necrosis factor (TNF-α), IL-4, IL-10, IL-13, fibroblast growth factor 2 (FGF-basic), and Monocyte chemoattractant protein-1 (MCP-1). Multivariable logistic regression models showed that advanced liver fibrosis was associated with an increased plasma level of IL-1β, IL-6, IL-7, IL-12, IL-17, GM-CSF, IFN-γ, IL-4, IL-10, MCP-1, Eotaxin, and FGF-basic, with FGF-basic continuing to be positively and significantly associated with advanced liver fibrosis, after Bonferroni correction for multiple comparisons (adjusted odds ratio (aOR) = 1.92; 95%CI: 1.32–2.81; *p* = 0.001). Plasma sCD14 was also significantly associated with advanced liver fibrosis (aOR = 1.13; 95%CI: 1.01–1.30; *p* = 0.049). Conclusions: HIV–HCV-coinfected patients are living with a high prevalence of advanced liver fibrosis which coexists with a mixture of elevated plasma inflammation and microbial translocation biomarkers. The significant associations of advanced liver fibrosis with FGF-basic and sCD14 may reveal pathogenic mechanisms and potential clinical intervention targets for liver fibrosis in HCV–HIV coinfection.

## 1. Introduction

Coinfection with human immunodeficiency virus (HIV) and hepatitis C virus (HCV) brings a heavy disease burden to the infected population and is a major public health concern worldwide [1]. HIV–HCV-coinfected patients have more frequent and accelerated progression to fibrosis and end-stage liver diseases (ESLD), including cirrhosis and hepatocellular carcinoma (HCC), compared to HCV monoinfected patients [2]. This is a serious public health challenge in China, given the fact that China has a large number of HIV–HCV-coinfected patients, who are most likely to be former plasma donors or injection drug users [3]. However, different from Western countries, direct-acting antivirals (DAAs) or even peg-interferon plus ribavirin (PR) for HCV treatment are not widely used in China, especially in rural areas, because of limited access to health care and the high prices of the drugs, which lead to a heavier disease burden [4]. The pathogenesis of progressive liver fibrosis in HIV–HCV coinfection is complex [5], and is mainly characterized by a higher HCV viral load, altered cellular immunity, and elevated pro-inflammatory and profibrogenic cytokines. More specifically, the immune suppression and cluster of differentiation 4 (CD4) T-cell depletion induced by HIV infection cause persistent immune activation, and Kupffer cells and hepatic stellate cells (HSCs) are directly stimulated to secrete pro-fibrotic cytokines or type 1 collagen [6]. In addition, HIV infection causes weakened CD4 and cluster of differentiation 8 (CD8) T-cell responses, leading to the persistence of HCV infection [7], which in turn contributes to the increased rates of liver fibrosis [8].

Moreover, microbial translocation (MT), featuring the dysfunction of the mucosal barrier and increased intestinal permeability due to immune dysregulation and intestinal microbiome change, has been commonly observed among HIV-infected individuals [9]. Microbial components including lipopolysaccharide (LPS), lipoteichoic acid, and flagellin may promote liver fibrosis by the stimulation of HSCs and Kupffer cells with a LPS binding protein (LBP) and soluble CD14 (sCD14). These microbial components may enhance hepatic inflammation, immune response, and liver cell death [5].

Thus, the above-mentioned mechanisms jointly emphasize the important role played by inflammation in liver fibrosis among HIV–HCV-coinfected patients. The cirrhosis-associated dysregulation of immune responses can be reflected by the increased production and elevated circulating levels of pro-inflammatory cytokines [10]. Kupffer cells generate IL-1β, IL-6, IL-12, and IL-18, and also release anti-inflammatory cytokines, including IL-10 stimulated by LPS [11]. The upregulation of IL-4, IL-13, transforming growth factor (TGF-) β1, and platelet-derived growth factor (PDGF) was observed during fibrogenesis, while glycoprotein (gp120) induces HSC accumulation by the secretion of monocyte chemoattractant protein-1 (MCP-1) by HSCs among HIV-infected patients [12]. Furthermore, HIV suppression by combination antiretroviral therapy (cART) may decrease inflammation and immune activation and may slow down the progression of liver disease [13]; however, the levels of plasma inflammatory biomarkers may remain abnormal in many individuals [14]. Thus far, the inflammatory profile among HIV–HCV-coinfected patients with different levels of liver fibrosis has not been well established, especially under the circumstance of combination antiretroviral therapy (cART). To fill this gap, the present study aimed to examine the association of advanced liver fibrosis with microbial translocation and inflammation cytokines among HIV–HCV-coinfected patients in the era of cART.

## 2. Materials and Methods

### 2.1. Study Population

The present cross-sectional study was conducted in Dehong Prefecture of Yunnan Province at China’s southwest border. Injection drug use (IDU) was the predominant mode of HIV transmission throughout the early 2000s, and continues to be an important mode of HIV and HCV infection [15]. By March 2016, 1017 HIV–HCV-coinfected patients, with 170 (16.7%) deaths, had been reported to and registered in the Comprehensive Response Information Management System (CRIMS) for HIV and AIDS in China [16]. Out of the remaining 847 patients, 463 (54.7%) were identifiable during the study period from April to October in 2016. Of them, 390 (84.2%) were receiving cART and gave informed consent to participate in the present study. Forty-seven patients were further excluded from the study due to missing data on biochemical measures for FIB-4 calculation, including aspartate aminotransferase (AST), alanine aminotransferase (ALT), and platelet count (PLT). Thus, a total of 343 HIV–HCV-coinfected patients were included in the final analysis. These participants showed no significant differences from the 120 identified yet excluded patients in the distribution of age, sex, marital status, HIV transmission route, hepatitis B surface antigen (HBsAg) serostatus, baseline CD4 cell count, years on cART, and current HCV RNA and HIV RNA levels, but showed a difference in ethnicity (Table S1). The study was approved by the Institutional Review Board of Fudan University School of Public Health, Shanghai, China (Approval number: IRB#2013-03-0407). All the subjects provided informed consent prior to enrollment.

### 2.2. Data Extraction

Demographical and clinical epidemiological data were extracted from the CRIMS. The data included age, sex, marital status, ethnicity, glucose level, HIV transmission route, date of cART initiation, cART regimen, CD4+ T cell counts at cART initiation, and follow-up visits.

### 2.3. Blood Testing

#### 2.3.1. Biochemical Tests for Liver Fibrosis Assessment

Liver biopsy is an invasive technique with complications and is costly, though it is a gold reference standard for determining liver fibrosis. In the light of these limitations, we assessed liver fibrosis by FIB-4 score, calculated as (AST [IU/L] × age [years])/(PLT [10^9^/L] × ALT[IU/L]^1/2^) using Sterling’s formula [17], which is an internationally recognized, well-established, noninvasive indicator for liver fibrosis [18]. Biochemical measurements of AST, ALT, and PLT were performed using an automatic biochemistry analyzer (Beckman Coultner, Brea, CA, USA), according to the manufacturer’s protocol.

FIB-4 is generally divided into three categories: no or mild liver fibrosis with FIB-4 < 1.45, intermediate liver fibrosis with 1.45 ≤ FIB-4 ≤ 3.25, and advanced liver fibrosis with FIB-4 > 3.25. In this study, we considered advanced liver fibrosis (FIB-4 > 3.25) as the major outcome.

#### 2.3.2. HCV RNA Quantification and Genotyping

Plasma HCV viral RNA was extracted (Roche diagnostic products Co., Ltd., Shanghai, China), and quantified by a real-time polymerase chain reaction (RT-PCR) technique using commercially available kits for the quantification of HCV RNA (PCR-Fluorescent Probing, PG Biotech Ltd., Shenzhen, China). The limit of detection was 500 copies/mL, and the linear range of HCV RNA quantification was from 1.0 × 10^3^ to 5.0 × 10^7^ copies/mL.

Amplification was completed by a nested PCR with E1- or NS5B-specific primers. Splicing, proofreading, and aligning sample sequences were performed using the ChromasPro 1.5 and BioEdit7.0.9.0 software. HCV genotype reference sequences were retrieved from the HCV database (http://hcv.lanl.gov/content/sequence/HCV/ToolsOutline.html). The HCV gene subtype was determined according to the phylogenetic tree, which was established by the neighbor-joining method using the MEGA 7.0 software [19].

#### 2.3.3. Multiplex Cytokine Bead Assay and sCD14

Twenty-seven cytokines, chemokines, and growth factors cytokines in plasma specimens were quantified by a multiplex analysis performed on the BioPlex^®^200 Multiplex System platform using the Bio-Plex Human 27-plex panel of cytokines, chemokines, and growth factors (Bio-Rad, Hercules, CA, USA), according to the manufacturer’s instructions. These biomarkers included (1) proinflammatory biomarkers such as interleukin (IL)-1β, IL-2, IL-6, IL-7, IL-8, IL-9, IL-12, IL-15, IL-17, granulocyte colony stimulating factor (G-CSF), granulocyte macrophage colony stimulating factor (GM-CSF), Interferon-γ (IFN-γ), and tumor necrosis factor (TNF-a); (2) anti-inflammatory biomarkers such as IL-1ra, IL-4, IL-5, IL-10, and IL-13; (3) chemokines such as IFN-γ-inducible protein (IP-10), monocyte chemoattractant protein-1 (MCP-1), macrophage inflammatory proteins-1alpha (MIP-1a), MIP-1b, and regulated upon activation normal T-cell expressed and secreted (RANTES); and (4) growth factors such as Eotaxin, fibroblast growth factor 2 (FGF-basic), platelet-derived growth factor (PDGF-BB), and vascular endothelial growth factor (VEGF). The detection limit for each molecule was determined by the recovery of the corresponding standard, and the lowest values with more than 70% recovery were set as the lowest detection limits. Measurements less than the lower limit of quantification (LLOQ) were assigned a value of half the LLOQ, and measurements more than the upper limit of quantification (ULOQ) were assigned a value of twice the ULOQ for each marker.

Plasma sCD14 was measured according to the manufacturer’s protocol for commercial enzyme-linked immunosorbent assay (ELISA) (Hycult Biotech, Wayne, PA, USA).

#### 2.3.4. Other Tests

Human hepatitis B virus surface antigen (HBsAg) was tested by the ELISA technique (Wan Tai Biomedical Co. Ltd., Beijing, China). The CD4 cell counts were assessed by FACSCount (Becton, Dickinson and Co., San Jose, CA, USA).

### 2.4. Statistical Analysis

The distributions of categorical variables were compared using chi-square test or Fisher’s exact test, and continuous variables were compared using a t-test or the Mann–Whitney U test. A log10-transformation was conducted for plasma cytokines levels for further statistical and regression analyses. A multiple logistic regression analysis, with adjustment for age (as a continuous variable), sex (0: male, 1: female), ethnicity (0: Han, 1: Dai, 2: Jingpo, 3: others, entered as dummy variables), current HIV RNA (0: undetectable, 1: detectable), current HCV RNA (0: undetectable, 1: detectable), current CD4 cell counts (0: < 200, 1-200-349, 2: ≥ 350, entered as dummy variables), glucose level (as a continuous variable), years on cART (as a continuous variable), and cART regimen type (0: NVP, 1: TDF, 2: others, entered as dummy variables), was used to explore the association of liver fibrosis with the plasma level of each of the 27 cytokines and sCD14. The odds ratio (OR) and 95% confidence interval (95%CI) represent the risk of higher FIB-4 with per one log-unit change in plasma cytokine concentration. Spearman correlations were computed to explore correlations between the plasma inflammatory biomarkers. Statistical significance was defined as *p* < 0.05 and Bonferroni *p* < 0.002 (0.05/27 ≈ 0.002) for multiple comparison adjustment. All the statistical analyses were performed using the R software (version 3.3.2, R Foundation for Statistical Computing, Vienna, Austria).

## 3. Results

### 3.1. Demographic and Clinical Characteristics

The median age of the participants was 35.4 (interquartile range (IQR): 31.3–39.3) years old. The majority of the participants were male (98%), had injection drug use (IDU) as the HIV transmission route (91.5%), were seronegative for hepatitis B surface antigen (HBsAg) (91.3%), and were detectable for plasma HCV RNA (69.4%) but undetectable for plasma HIV RNA (89.3%). Around half of the participants were non-Han ethnicities—i.e., ethnic minorities (54.2%); currently married (51.9%); and of HCV subtype 3 (47%) (Table 1).

### 3.2. Prevalence of Advanced Liver Fibrosis

Among all the participants, 75 (21.9%) had no or mild liver fibrosis (FIB-4), 80 (23.3%) had intermediate liver fibrosis (FIB-4), and 188 (54.8%) had severe or advanced liver fibrosis (FIB-4 > 3.25). Compared to patients without advanced liver fibrosis, those with advanced liver fibrosis were older, under a different cART regimen, and had a longer duration on cART (Table 1).

### 3.3. Plasma Levels of Inflammatory Cytokines

**Figure 1:**
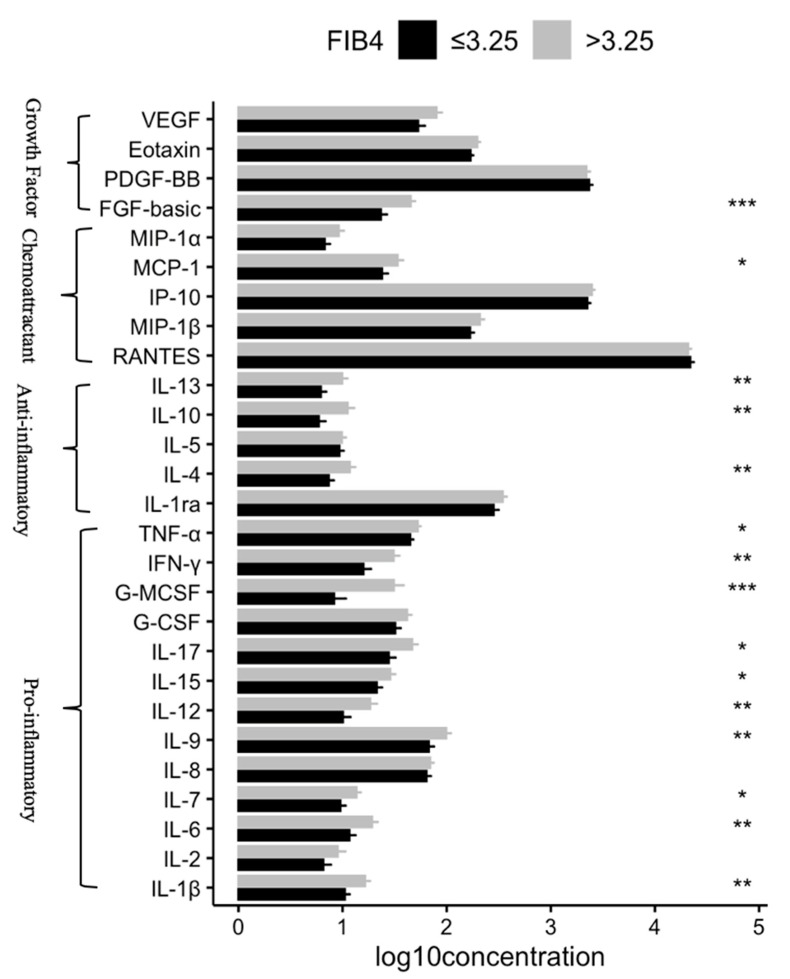
Comparison of log10-transformed plasma concentration of tested biomarkers between participants with different FIB-4 scores. * *p* < 0.05, ** *p* < 0.01, and *** *p* < 0.001. All the *p*-values were calculated by Mann–Whitney tests.

Participants with sever liver fibrosis (FIB-4 > 3.25) had significantly higher plasma levels of IL-1β, IL-4, IL-6, IL-7, IL-9, IL-10, IL-12, IL-13, IL-15, IL-17, IFN-γ, TNF-α, MCP-1, GM-CSF, FGF-basic, and VEGF than those with FIB-4 ≤ 3.25 (all *p* < 0.05) (Table 2 and Figure 1). Such differences remained significant for IL-6, IL-10, IFN-γ, GM-CSF, and FGF-basic after Bonferroni correction for multiple comparisons (all *p* ≤ 0.002 or 0.05/27). No significant difference in the sCD14 level was observed between the two groups.

### 3.4. Correlations of Inflammatory Cytokines with Advanced Liver Fibrosis

**Figure 2:**
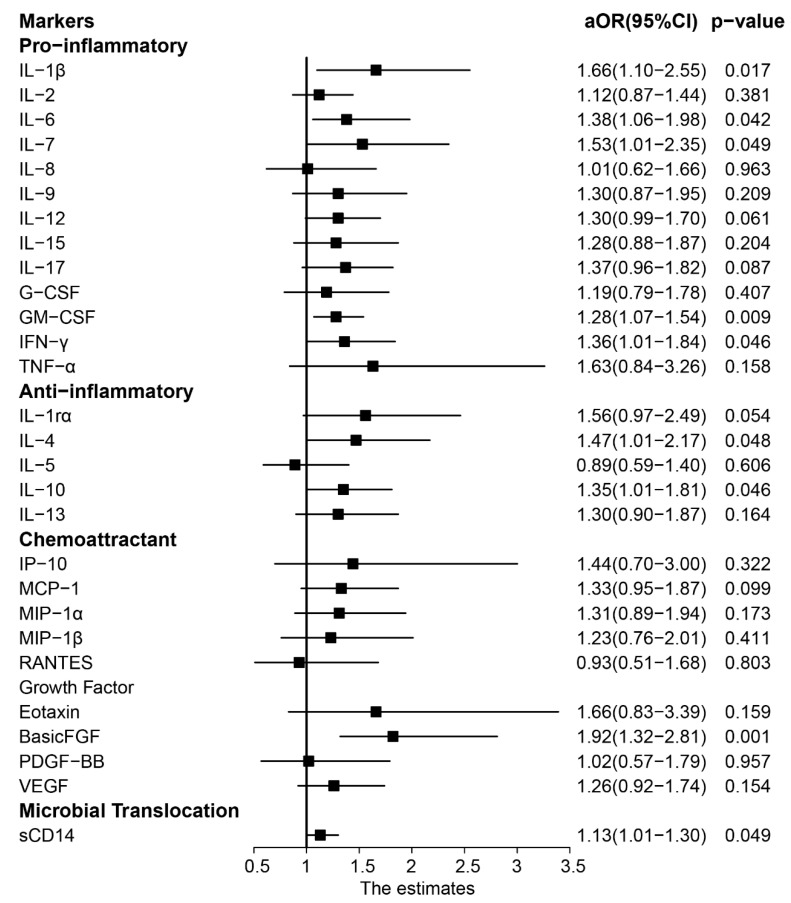
Adjusted odds ratios and 95% confidence intervals for the associations between plasma cytokine levels (log10-transformed) and advanced liver fibrosis among HIV–HCV-coinfected patients. Each association was assessed in a separate multivariable logistic regression model adjusting for age, sex, ethnicity, glucose level, current HIV RNA, current HCV RNA, current CD4 count, years on cART, and cART regimen.

Multivariable logistic regression adjusting for potential confounders was performed to examine the association of advanced liver fibrosis with each of the 27 cytokines and chemokines and sCD14 levels. Advanced liver fibrosis was shown to be associated with higher levels of 11 out of the 27 markers (*p* < 0.05), while FGF-basic continued to be positively and significantly associated with advanced liver fibrosis after Bonferroni correction for multiple comparisons (aOR = 1.92; 95%CI: 1.32–2.81; *p* = 0.001). Furthermore, the plasma sCD14 was significantly associated with advanced liver fibrosis (aOR = 1.13; 95%CI: 1.01–1.30; *p* = 0.049, Figure 2).

## 4. Discussion

In the present study, 54.8% of the HIV–HCV-coinfected patients had advanced liver fibrosis. The prevalence was much higher than in studies conducted in Western countries, which ranged from 22.9% to 40.3% [20,21,22].

HIV and HCV infections are both characterized by systemic inflammation with increased levels of blood inflammatory cytokines. Although cART might help reduce the plasma inflammatory marker levels in HIV–HCV-coinfected patients, the markers may still remain much higher than healthy controls [23]. In line with our findings, elevated liver fibrosis was previously linked to increased biomarkers of inflammation in HIV–HCV-coinfected patients [24,25]. In the present study, we identified a mixed profile of elevated circulating inflammatory cytokines, chemokines, and growth factors, including IL-6, IL-10, IFN-γ, GM-CSF, and FGF-basic, among HIV–HCV-coinfected patients with advanced liver fibrosis. This finding further indicates that the main syndrome of HIV–HCV-coinfected patients with progressive and advanced liver fibrosis is inflammation [12].

In multiple logistic regression analyses, adjusting for potential confounders, FGF-basic was shown to be significantly and positively associated with advanced liver fibrosis upon Bonferroni correction for multiple comparisons. FGF-basic is a single-chain polypeptide growth factor that plays a significant role in the process of wound healing, is a potent inducer of angiogenesis, and has potent heparin-binding activity. Recent studies have suggested potential yet controversial associations of FGF-basic with disease status among HIV patients [26,27]. The present finding of the positive association between FGF-basic and advanced liver fibrosis may reveal a pathogenic mechanism and a potential clinical intervention target for liver fibrosis in HCV–HIV coinfection.

The significant associations of advanced liver fibrosis with higher levels of IL-6, IFN-γ, IL-10, and GM-CSF, despite being insignificant upon Bonferroni correction, are in accordance with some previous studies and are worthy of further investigation [28,29,30,31]. IL-6 is profibrogenic and may induce liver damage [32]. The role of IFN-γ in liver fibrosis is controversial. In some studies, IFN-γ was negatively correlated with liver fibrosis [33], while IFN-γ was also found to have proinflammatory effects that can aggravate disease progression and organ dysfunction [30], and an increased production of IFN-γ is observed among cirrhotic patients [34]. Soluble IL-10 was higher in HIV–HCV-coinfected patients than in HIV-negative persons [27], but could be reduced following peg-interferon plus ribavirin (PR) treatment for HCV [35]. GM-CSF was higher in HIV patients than in healthy control subjects [31], and may promote liver damage and liver fibrosis [36]. sCD14 is often employed as an indirect biomarker of microbial translocation, given the technical difficulty in measuring lipopolysaccharide (LPS), which is a major marker of microbial translocation [37]. Our study shows that the sCD14 level was positively associated with advanced liver fibrosis, consistent with the previous studies [38,39], further suggesting the role of inflammation in liver complications in HIV–HCV patients. sCD14 reflects the intestinal flora entering the blood or means the activation of secondary inflammation, and FGF-basic increases the permeability of epithelial cells. These suggest that regulating the flora and even controlling inflammation will help prevent or slow down the progression of severe liver fibrosis.

It is worth noting that chronic hepatitis C with severe fibrosis is associated with insulin resistance/diabetes [40] and oxidative stress [41]. We found that the plasma glucose levels are correlated with IL-1β, IL-6, MIP-1α, MIP-1β, and VEGF (data not shown).

This study had several limitations. First, no causal relationship could be drawn upon the cross-sectional design of this study. Second, data are unavailable on participants’ alcohol consumption, which is known to influence the risk of liver fibrosis and could alter the levels of immune and inflammatory markers. Thirdly, because of a lack of lipid peroxidation and oxidative stress measurements, we could not examine the correlation of inflammatory biomarkers with the oxidative stress parameter. This should be addressed in future research.

## 5. Conclusions

In summary, HIV–HCV-coinfected patients are living with a high prevalence of advanced liver fibrosis which coexists with a mixture of elevated plasma inflammation and microbial translocation biomarkers. The significant associations of advanced liver fibrosis with FGF-basic and sCD14 may reveal pathogenic mechanisms and potential clinical intervention targets for liver fibrosis in HCV–HIV coinfection.

## Figures and Tables

**Table 1:** Demographic and clinical characteristics of the HIV–HCV-coinfected participants.

Characteristics	All No. (%)	FIB-4 Score		*p* ^a^
		≤3.25 No. (%)	>3.25 No. (%)	
Overall	343 (100.0)	155 (45.2)	188 (54.8)	
Age, years				
Median (IQR)	35.4 (31.3–39.3)	34.6 (30.2–38.6)	36.0 (31.8–40.3)	**0.029**
Sex				
Male	336 (98.0)	151 (97.4)	185 (98.4)	0.796
Female	7 (2.0)	4 (2.6)	3 (1.6)	
Marital status				0.210
Unmarried	130 (37.9)	61 (39.4)	69 (36.7)	
Married	178 (51.9)	74 (47.7)	104 (55.3)	
Divorced/widowed	35 (10.2)	20 (12.9)	15 (8.0)	
Ethnicity				0.151
Han	157 (45.8)	62 (40.0)	95 (50.5)	
Dai	112 (32.6)	60 (38.7)	52 (27.7)	
Jingpo	62 (18.1)	28 (18.1)	34 (18.1)	
Others	12 (3.5)	5 (3.2)	7 (3.7)	
HIV transmission route				0.185
IDU	314 (91.5)	140 (90.3)	174 (92.6)	
Others	29 (8.5)	15 (9.7)	14 (7.4)	
Glucose level (IQR)	5.20 (4.32–6.00)	5.29 (4.31–6.03)	5.16 (4.37–5.89)	0.489
Baseline CD4, cells/ul				0.618
<200	98 (29.0)	40 (26.3)	58 (31.2)	
200–349	139 (41.1)	65 (42.8)	74 (39.8)	
≥350	101 (29.9)	47 (30.9)	54 (29.0)	
Current CD4, cells/ul				0.511
<200	21 (6.1)	7 (4.5)	14 (7.4)	
200–349	52 (15.2)	23 (14.8)	29 (15.4)	
≥350	270 (78.7)	125 (80.6)	145 (77.1)	
HCV RNA				0.234
Undetectable	105 (30.6)	53 (34.2)	52 (27.7)	
Detectable	238 (69.4)	102 (65.8)	136 (72.3)	
HIV RNA				0.053
Undetectable	264 (89.3)	126 (88.1)	138 (79.3)	
Detectable	53 (10.7)	17 (11.9)	36 (20.7)	
Years on cART	5.7 (4.2–8.4)	5.1 (3.9–7.6)	6.1 (4.4–8.9)	**0.001**
HBsAg positive				0.685
Yes	30 (8.7)	12 (7.7)	18 (9.6)	
No	313 (91.3)	143 (92.3)	170 (90.4)	
cART regimen, %				**0.012**
NVP (vs. EFV/RTV)	136 (39.7)	52 (33.5)	84 (44.7)	
TDF (vs. AZT/d4T/DDI)	147 (42.9)	80 (51.6)	67 (35.6)	
Others	60 (17.4)	23 (14.8)	37 (19.7)	
HCV genotype				0.182
1	46 (16.0)	23 (18.1)	23 (14.4)	
3	135 (47.0)	52 (40.9)	83 (51.9)	
6	106 (37.0)	52 (40.9)	54 (33.8)	

IDU, injection drug use; NVP, nevirapine; EFV, efavirenz; RTV, Ritonavir; TDF, tenofovir; AZT, zidovudine; DDI, di-deoxyinosine; d4T, stavudine; ^a^ Chi-square tests or the Mann–Whitney U test were performed wherever appropriate; bold signifies statistical significance as *p* < 0.05.

**Table 2:** Plasma level of inflammatory cytokines according to FIB-4 score.

	Overall	FIB-4 Score		*p*
		≤3.25	>3.25	
Pro-inflammatory				
IL-1β	9.8 (5.0, 46.9)	8.0 (4.6, 33.8)	11.3 (5.2, 63.0)	0.005
IL-2	6.9 (0.50, 87.0)	6.1 (0.5, 63.4)	9.6 (0.5, 95.6)	0.216
IL-6	19.8 (6.8, 46.3)	16.3 (4.8, 37.0)	23.6 (10.2, 54.6)	0.001
IL-7	14.4 (6.2, 29.8)	13.2 (5.3, 24.6)	15.6 (6.4, 35.0)	0.038
IL-8	63.6 (32.5, 123.9)	56.0 (31.7, 117.5)	73.0 (34.5, 123.8)	0.166
IL-9	92.8 (39.4, 267.5)	81.5 (32.7, 183.4)	103.0 (50.0, 337.1)	0.009
IL-12	27.0 (1.9, 73.0)	20.9 (1.5, 58.3)	32.4 (3.7, 93.5)	0.006
IL-15	23.0 (5.3, 112.1)	14.8 (5.3, 86.4)	27.6 (5.3, 127.0)	0.046
IL-17	92.2 (11.1, 146.6)	46.0 (9.7, 129.0)	106.3 (13.8, 154.1)	0.016
G-CSF	52.0 (27.2–82.0)	44.5 (20.7, 89.7)	54.8 (36.1, 80.1)	0.278
GM-CSF	93.7 (0.3, 238.3)	17.6 (0.2, 196.2)	121.0 (2.2, 269.3)	**<0.001**
IFN-γ	41.0 (13.8, 72.4)	29.9 (5.0, 65.7)	47.0 (26.9, 75.2)	**0.002**
TNF-α	46.0 (31.1, 70.0)	40.8 (27.1, 70.3)	50.5 (35.6, 68.3)	0.015
Anti-inflammatory				
IL-1rα	324.5 (141.0, 755.4)	288.9 (124.1, 722.8)	338.1 (156.0, 801.7)	0.210
IL-4	5.6 (2.9, 61.0)	5.1 (2.6, 11.2)	5.9 (3.3, 80.0)	0.008
IL-5	10.0 (4.5, 22.0)	10.6 (4.3, 20.0)	10.0 (4.7, 23.0)	0.632
IL-10	9.9 (0.7, 61.0)	7.4 (0.7, 40.8)	11.8 (0.7, 71.6)	**0.001**
IL-13	8.4 (3.0, 32.0)	6.6 (2.1, 20.9)	10.1 (3.7, 34.1)	0.006
Chemoattractant				
IP-10	2390.3 (1331.0, 4196.2)	2322.5 (1244.2, 4004.6)	2565.7 (1492.8, 4208.3)	0.335
MCP-1	46.1 (14.9, 88.2)	32.9 (14.2, 77.0)	53.2 (16.0, 112.9)	0.013
MIP-1α	4.9 (3.0, 37.0)	5.1 (2.6, 19.2)	4.8 (3.4, 42.3)	0.102
MIP-1β	132.2 (84.1, 410.5)	126.0 (87.6, 235.9)	142.5 (80.4, 551.3)	0.229
RANTES	19,871.0 (11759.0, 69279.6)	19,700.5 (9854.2, 69279.6)	19,979.5 (13163.8, 69279.6)	0.881
Growth factor				
Eotaxin	182.7 (117.7, 294.1)	179.1 (117.6, 263.7)	193.0 (118.6, 324.2)	0.126
FGF-basic	66.0 (9.8, 104.5)	40.9 (4.35, 97.1)	79.5 (30.6, 107.0)	**0.001**
PDGF-BB	2840.7 (1266.9, 4561.9)	2750.9 (1323.52, 4295.5)	2924.1 (1231.3, 4616.9)	0.978
VEGF	105.0 (27.05, 215.1)	94.0 (7.6, 196.9)	111.7 (47.0, 230.5)	0.050
Microbial Translocation				
sCD14	3.0 (2.2, 4.2)	2.9 (2.2, 3.9)	3.2 (2.2, 4.7)	0.094

Data were presented as median and interquartile range (IQR), with μg/mL as the unit for sCD14 and pg/mL for all other cytokines. All the *p*-values were calculated by Mann–Whitney tests. Bold signifies statistical significance as *p* < 0.05.

## Supplementary Materials

The following are available online at https://www.mdpi.com/1660-4601/17/24/9474/s1: Table S1: Comparison of baseline characteristics between included and excluded group.
