# Supplementary material for: Plasma Inflammatory Biomarkers Associated with Advanced Liver Fibrosis in HIV–HCV-Coinfected Individuals
Source: Int J Environ Res Public Health. 2020 Dec 17;17(24):9474. doi: 10.3390/ijerph17249474 (PMC7766690; doi:10.3390/ijerph17249474)
Supplement: Supplementary file 1 [file ijerph-17-09474-s001.pdf]

Table S1. Comparison of baseline characteristics between included and excluded group.

	Total	Excluded	Included	*p*
N	463	120	343	
Age, year				
Median (IQR)	31.53 ± 7.16	31.53 ± 7.31	31.53 ± 7.11	0.995
Gender				0.999
Male	454 (98.1)	118 (98.3)	336 (98.0)	
Female	9 (1.9)	2 (1.7)	7 (2.0)	
Marital status				0.398
Unmarried	181 (39.3)	51 (43.6)	130 (37.9)	
Married	229 (49.8)	51 (43.6)	178 (51.9)	
Divorced/widowed	50 (10.9)	15 (12.8)	35 (10.2)	
Ethnicity				**<0.001**
Han	204 (44.3)	47 (40.2)	157 (45.8)	
Dai	140 (30.4)	28 (23.9)	112 (32.7)	
Jingpo	79 (17.2)	17 (14.5)	62 (18.1)	
Others	37 (8.0)	25 (21.4)	12 (3.5)	
HIV transmission route				
IDU	421 (92.5)	107 (95.5)	314 (91.5)	0.235
Others	42 (7.5)	13 (4.5)	29 (8.5)	
Glucose level	5.12(4.23–6.02)	5.25(4.19-6.10)	5.20 (4.32–6.00)	0.355
Baseline CD4, cells/ul				0.243
<200	114 (27.6)	16 (21.3)	98 (29.0)	
200-349	169 (40.9)	30 (40.0)	139 (41.1)	
≥350	130 (31.5)	29 (38.7)	101 (29.9)	
As continuous	275 (192–408)	293 (220–437)	270 (188–399)	0.167
Baseline FIB-4				0.846
<1.45	204 (49.9)	35 (47.3)	169 (50.4)	
1.45-3.25	120 (29.3)	22 (29.7)	98 (29.3)	
>3.25	85 (20.8)	17 (23.0)	68 (20.3)	
As continuous	1.75 (1.17–2.90)	1.82 (1.27–3.02)	1.75 (1.17–2.86)	0.993
HBsAg positive				0.998
Yes	40 (8.6)	10 (8.3)	30 (8.7)	
No	423 (91.4)	110 (91.7)	313 (91.3)	
ART regimen type				0.671
NVP (vs EFV/RTV)	162 (38.7)	26 (34.2)	136 (39.7)	
TDF (vs AZT/d4T/DDI)	182 (43.4)	35 (46.1)	147 (42.9)	
Others	75 (17.9)	15 (19.7)	60 (17.5)	
